# Predictors of Resistance in Pediatric *Helicobacter pylori* Infection

**DOI:** 10.3390/pathogens15060608

**Published:** 2026-06-05

**Authors:** Kim Ruiz-Arellanos, Maria Camila Cardenas-Fernández, Silvana Bonilla

**Affiliations:** 1 Department of Pediatrics, Boston Children’s Hospital, Boston, MA 02115, USA; 2 Division of Pediatric Gastroenterology, Hepatology, and Nutrition, Boston Children’s Hospital, Boston, MA 02115, USA

**Keywords:** antimicrobial resistance, children, metronidazole, clarithromycin

## Abstract

*Helicobacter pylori* (*H. pylori*) antimicrobial resistance is a key driver of eradication failure in children and adults. Recognizing factors associated with *H. pylori* antimicrobial resistance may help identify individuals at risk for treatment failure and tailor management. We conducted a retrospective cohort study of patients with *H. pylori*-positive gastric biopsy culture and available antimicrobial susceptibility data at a large pediatric tertiary care center between 2020 and 2025. Descriptive statistics and logistic regression models were used to assess associations among demographic characteristics, prior treatment history, and antimicrobial resistance. Of 174 patients (56.1% male, median 14 years), 50.3% were resistant to at least one antimicrobial. Resistance rates included metronidazole (28.3%), clarithromycin (18.5%), rifampin (12.7%), fluoroquinolones (11.6%), amoxicillin (4%), and tetracycline (0.6%). The multidrug resistance rate was 17.3%. Prior *H. pylori* treatment was the most consistent predictor of antimicrobial resistance on both bivariate and multivariable analyses. Race was independently associated with metronidazole resistance, with Black patients exhibiting the highest rates and significantly greater odds compared to White patients. Hispanic ethnicity was also independently associated with increased odds of metronidazole resistance. Careful consideration of prior treatment history, race, and ethnicity is warranted when managing pediatric *H. pylori* infection, given their association with increased antimicrobial resistance.

## 1. Introduction

*Helicobacter pylori* (*H. pylori*) infection rates have declined over recent decades; however, it continues to affect a large proportion of the global population [1]. Although infected individuals, particularly children, are often asymptomatic, the infection is associated with well-documented complications, including chronic gastritis, peptic ulcer disease, and, less commonly, gastric cancer [2]. Eradication therapy for *H. pylori* consists of acid-suppressive medication combined with two to three antimicrobials, with or without bismuth (triple or quadruple therapy), yet eradication rates have declined over time [3,4,5].

Antimicrobial resistance is considered one of the most important factors linked to *H. pylori* eradication failure in children and adults [6,7,8]. A systematic review and meta-analysis of pediatric populations over the past two decades, spanning 28 countries across five World Health Organization (WHO) regions, reported high resistance rates to metronidazole (35.3%), clarithromycin (32.6%), and levofloxacin (13.2%), while resistance to amoxicillin and tetracycline remained low at 4.8% and 2.1%, respectively [8]. Similar resistance patterns have been reported in adult populations, with rates of clarithromycin, metronidazole, and levofloxacin resistance far exceeding the 15% threshold recommended for empirical use [6,7].

Pediatric guidelines recommend antimicrobial susceptibility testing to guide therapy, particularly for clarithromycin [5,9,10]. However, the recommended methods, gastric biopsy culture or PCR, are not widely available. Institutional efforts to standardize gastric biopsy culture protocols have demonstrated that targeted educational interventions and process improvements can significantly increase culture yield, supporting broader implementation of susceptibility-guided therapy [11]. In the absence of susceptibility data, clinicians may benefit from considering patient characteristics that influence treatment outcomes and inform therapy selection. Factors such as prior antimicrobial exposure, age, race, ethnicity, geographic location, bacterial virulence, and endoscopic findings of peptic ulcer disease have been associated with resistance patterns in adults, though data in pediatric populations remain limited [12,13,14,15,16,17,18,19,20,21]. This study aims to build on prior pediatric research examining associations between clinical characteristics and antimicrobial resistance.

## 2. Materials and Methods

### 2.1. Study Design

This is a retrospective cohort study of patients with *H. pylori*-positive gastric biopsy culture and available antimicrobial susceptibility data at a large tertiary care center (Boston Children’s Hospital, Boston, MA, USA), between January 2020 and December 2025. Patients who lacked antimicrobial susceptibility data were excluded from the study.

### 2.2. Antimicrobial Susceptibility Testing

In accordance with institutional protocol, gastric biopsy samples (1 from the antrum and 1 from the corpus) were obtained during endoscopy, placed in a sterile container on ice, and sent to a reference laboratory (Mayo Clinic Laboratories, Rochester, MN, USA) for *H. pylori* culture and susceptibility testing [11]. When *H. pylori* were isolated, organism identification was confirmed and antimicrobial susceptibility testing was performed. The routine susceptibility panel included amoxicillin, clarithromycin, levofloxacin, metronidazole, rifampin, and tetracycline. Mayo Clinic Laboratories performed susceptibility testing using validated agar dilution methodology and established interpretive criteria. Minimal inhibitory concentrations (MICs) were determined by the reference laboratory and interpreted using predefined susceptibility breakpoints (amoxicillin ≤0.008 mcg/mL, clarithromycin ≤0.25 mcg/mL, metronidazole ≤8 mcg/mL, tetracycline ≤1 mcg/mL, rifampin ≤0.5 mcg/mL, and levofloxacin ≤1 mcg/mL). For the purposes of this study, individual MIC values were not analyzed; only the final susceptible/resistant interpretations reported by Mayo Clinic Laboratories were used in the analysis [22].

### 2.3. Data Collection

Extracted clinical data from the electronic medical records included patient demographic variables and prior *H. pylori* treatment history. Antibiotic exposure for non-*H. pylori* treatment purposes was not consistently reported and thus not included. Race and ethnicity were defined using U.S. Census Bureau categories: Black or African American, White, Asian, American Indian or Alaska Native, Native Hawaiian or Other Pacific Islander, and Other; ethnicity was categorized as Hispanic or Latino or Not Hispanic or Latino [23]. These categories are not mutually exclusive, as individuals of Hispanic ethnicity may identify with any racial group.

### 2.4. Statistical Analysis

Descriptive statistics were used to summarize patient characteristics, reported as median (interquartile range) for continuous variables and frequency (percentage) for categorical variables. Multivariable logistic regression models were fit for each antibiotic with a sufficient number of resistant cases (clarithromycin, metronidazole, fluoroquinolone, and rifampin; *n* ≥ 15 resistant cases each) to reduce the risk of model instability and overfitting. Amoxicillin and tetracycline were not included in multivariable models due to the low number of resistant isolates (*n* = 7 and *n* = 1, respectively), which limited the ability to generate stable estimates. All multivariable models included age (continuous), sex, race, Hispanic ethnicity, and prior *H. pylori* treatment as covariates, selected a priori based on clinical relevance. Results are reported as odds ratios (ORs) with 95% confidence intervals (CIs). Pairwise correlations between antibiotic resistances were assessed using Phi coefficients, a measure of association between binary variables. All tests were two-sided with a significance threshold of *p* < 0.05. Analyses were performed using R version 4.5.0 (R Foundation for Statistical Computing, Vienna, Austria).

The study was approved by the Boston Children’s Hospital Institutional Review Board (IRB).

## 3. Results

### 3.1. Population Characteristics

A total of 174 patients were included in the study. The median age was 14 years (IQR 9–16) and 56.1% were male. The cohort was racially diverse; racial distribution was as follows: 40.5% White, 22.0% Black or African American, 19.7% Asian, and 17.9% Other. Overall, 38.7% of patients identified as Hispanic (Table 1).

### 3.2. Antimicrobial Resistance Prevalence

**Figure 1:**
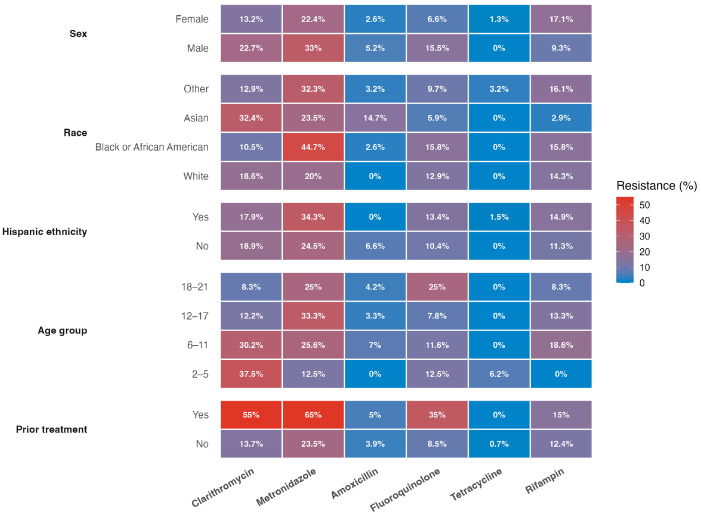
*H. pylori* Antibiotic Resistance Prevalence by Demographic Characteristics. Values represent the percentage of resistant isolates within each subgroup. *n* = 174 pediatric patients.

At least one antibiotic resistance was identified in 87 patients (50.3%), and multi-drug resistance (resistance to ≥2 antibiotics) was present in 30 patients (17.3%). Resistance rates were as follows: metronidazole (28.3%), clarithromycin (18.5%), rifampin (12.7%), fluoroquinolones (11.6%), amoxicillin (4%), and tetracycline (0.6%). Resistance prevalence stratified by demographic characteristics is summarized in Figure 1.

Among the 30 patients with multi-drug resistance, the most common co-resistance pattern was dual resistance to clarithromycin and metronidazole (*n* = 16, 53.3%), followed by metronidazole and fluoroquinolone (*n* = 14, 46.7%). These patterns are clinically significant, as they involve the two antimicrobials most commonly used in standard empiric triple therapy regimens.

To assess co-resistance patterns, pairwise Phi coefficients were calculated between all antibiotic pairs. All coefficients were below 0.35, indicating weak correlations between resistances to different antimicrobials (Supplementary Figure S1). The strongest co-resistance associations were observed between fluoroquinolone and metronidazole (φ = 0.33) and between clarithromycin and fluoroquinolone (φ = 0.25).

Resistance patterns were evaluated descriptively across the 2020–2025 study period. Persistent resistance to clarithromycin and metronidazole was observed throughout the study period, while resistance to fluoroquinolones, rifampin, and amoxicillin was less common but remained detectable over time. Temporal trend interpretation should be made cautiously, as the number of patients undergoing culture and susceptibility testing increased during the study period because of a concurrent institutional quality improvement initiative [11].

### 3.3. Age

Overall resistance rates were highest in the youngest age groups (37.5% in ages 2–5 and 30.2% in ages 6–11) compared to older groups (12.2% in ages 12–17 and 8.3% in ages 18–21). Younger age was significantly associated with clarithromycin resistance on both bivariate (*p* = 0.009) and multivariable analysis (OR 0.88 per year, 95% CI 0.80–0.97, *p* = 0.012), indicating that older children had lower odds of clarithromycin resistance. Age was not significantly associated with resistance to any other antibiotic (Table 2 and Table 3).

### 3.4. Sex

No significant associations were identified between sex and resistance to any antibiotic.

### 3.5. Race and Ethnicity

On bivariate analysis, race was significantly associated with metronidazole resistance (*p* = 0.048), with the highest rates observed among Black or African American patients (44.7%) compared to White patients (20.0%). This association remained significant on multivariable analysis, with Black or African American patients having significantly higher odds of metronidazole resistance compared to White patients (OR 5.73, 95% CI 2.08–17.1, *p* = 0.001) (Table 3).

Race was also significantly associated with amoxicillin resistance on bivariate analysis (*p* = 0.004), driven by a higher proportion of resistant isolates among Asian patients (71.4% of amoxicillin-resistant cases); however, given the small number of resistant cases (*n* = 7), these findings should be interpreted with caution, and no multivariable model was fit for this antibiotic (Table 2 and Table 3).

Race, ethnicity, and age were not significantly associated with fluoroquinolone or rifampin resistance in adjusted analyses. Hispanic ethnicity was independently associated with metronidazole resistance on multivariable analysis (OR 2.45, 95% CI 1.03–6.12, *p* = 0.047) (Table 3).

### 3.6. Prior *H. pylori* Treatment

Prior *H. pylori* treatment was the most consistent predictor of antimicrobial resistance across multiple antibiotics. On bivariate analysis, prior treatment was significantly associated with resistance to clarithromycin (34.4% vs. 13.7%, *p* < 0.001), metronidazole (65.0% vs. 23.5%, *p* < 0.001), and fluoroquinolone (35.0% vs. 8.5%, *p* = 0.003). These associations remained significant after multivariable adjustment. Patients with prior treatment had significantly higher odds of clarithromycin resistance (OR 7.58, 95% CI 2.63–23.1, *p* < 0.001), metronidazole resistance (OR 6.85, 95% CI 2.42–21.2, *p* < 0.001), and fluoroquinolone resistance (OR 5.36, 95% CI 1.66–16.9, *p* = 0.004) compared to treatment-naive patients (Table 2 and Table 3).

## 4. Discussion

We found high rates of antimicrobial resistance in our cohort of patients from a large pediatric tertiary care center in a low-prevalence region and identified factors associated with antimicrobial resistance. Our study adds to the existing literature on pediatric *H. pylori* infection and has important implications for clinical decision-making, particularly in supporting susceptibility-guided therapy and highlighting the need to consider demographic and regional resistance patterns when selecting empiric treatment regimens. By focusing on factors associated with antimicrobial resistance, this study provides clinically relevant data that may help optimize treatment strategies and improve eradication outcomes in pediatric patients.

Metronidazole and clarithromycin were the antimicrobials with the highest resistance rates, 28.3% and 18.5%, respectively, exceeding the 15% threshold recommended for use in empiric regimens [7]. These results align with pediatric data from pediatric registries in other low-prevalence regions, including Europe, where resistance rates of up to 28.8% for clarithromycin and 21% for metronidazole have been reported [13,24]. Notably, country-specific analyses demonstrate substantially higher rates, with clarithromycin resistance reported at 45% in Spain, 47% in Poland, and as high as 85% in Turkey in a recent pediatric systematic review and meta-analysis [8]. Similar patterns are observed for metronidazole. Notably, amoxicillin and tetracycline resistance rates remained below 5%, consistent with prior reports, supporting their continued inclusion in guideline-recommended empiric treatment regimens [5].

For antimicrobials less used in pediatric treatment regimens, fluoroquinolone resistance was 11.6%, exceeding rates reported in most European countries, except for Turkey (14%) and Bulgaria (19%) [8]. Possible reasons include higher exposure to antimicrobials for non-*H. pylori* indications in our selected tertiary care center population. This may also explain the relatively high rifampin resistance rate of 12.7%, which is not routinely reported in pediatric studies. This finding is novel and concerning, as treatment options are becoming increasingly limited. Newer rifamycin-based regimens (e.g., amoxicillin, rifabutin, and omeprazole), endorsed as first-line therapy in adult guidelines for *H. pylori* infection [4], may have reduced effectiveness in the setting of increasing rifampin resistance.

Younger age was significantly associated with clarithromycin resistance, with higher rates observed in children aged 2–11 years compared to older children. A pediatric meta-analysis found variation in global antimicrobial resistance rates according to both age group and antibiotic [8]. The highest clarithromycin resistance rate was observed among children aged 0–5 years (51.0%). These findings may be explained by several factors. Differences in immune system maturation across pediatric age groups may influence susceptibility to specific *H. pylori* strains and patterns of antimicrobial resistance. In addition, age-related variations in gut microbiota composition and prior antibiotic exposure may also contribute to differences in resistance patterns observed among children. The findings also may reflect local prescribing patterns and regional logistical factors, such as medication availability and access to compounded formulations, as well as provider reluctance to move away from clarithromycin-containing regimens in younger children [25]. These findings underscore the need for greater awareness and adherence to current guideline recommendations to defer treatment in young children when appropriate. This approach is particularly important given the unique considerations in pediatric patients, including medication tolerance and adherence challenges. In addition, unnecessary or repeated antibiotic exposure may contribute to secondary antimicrobial resistance, risk of reinfection, and disruption of the developing gut microbiota, all of which should be carefully weighed when making treatment decisions [5,26].

Resistance patterns varied across racial and ethnic groups. These findings should be interpreted cautiously, as race and ethnicity likely reflect differences in environmental exposures, prior antibiotic use, healthcare access, and other social determinants of health rather than biological determinants of resistance. We observed higher rates of metronidazole resistance among Black children. Similar demographic differences in antimicrobial resistance patterns have been reported in prior U.S. studies in pediatric *H. pylori* infection. One study identified Black race as a risk factor for resistance to at least one antibiotic [14], whereas others reported a lower likelihood of clarithromycin resistance in a cohort from two large pediatric tertiary care centers [21]. These differences may reflect variations in prior antibiotic exposure within specific tertiary care populations. We found a higher proportion of amoxicillin-resistant isolates among Asian patients, although the number of resistant cases was small. Previous studies have reported higher rates of clarithromycin resistance in Asian patients compared to non-Asian patients [21,27]. Additionally, Hispanic ethnicity was independently associated with metronidazole resistance. Potential explanations include increased antimicrobial exposure related to healthcare settings, antibiotic prescribing practices in countries of origin, self-medication practices, and differences in circulating *H. pylori* strains and bacterial virulence factors [28,29,30,31,32]. Socioeconomic and environmental factors, including healthcare access and prior antibiotic utilization, may also contribute to these observed disparities. Although molecular, physiologic, or host genetic factors could potentially influence susceptibility to resistant *H. pylori* strains, data evaluating these mechanisms in pediatric populations remain limited. Further multicenter studies with larger and more diverse patient populations are needed to confirm these associations and better characterize the role of social determinants of health in *H. pylori* resistance patterns.

Prior *H. pylori* treatment was the most consistent predictor of antimicrobial resistance across multiple antibiotics, consistent with the available pediatric literature [5]. Pediatric patients may be particularly vulnerable to the development of resistance because treatment adherence can be challenging due to medication intolerance and complex multidrug regimens such as bismuth quadruple therapy, which is the guideline-recommended empiric treatment regimen. To help mitigate the growing problem of antimicrobial resistance, extending the diagnostic evaluation by 7–10 days to obtain culture and susceptibility results before initiating therapy may be beneficial. This approach may be appropriate for most children, who rarely require immediate treatment initiation. Although this approach may delay the start of treatment and create some inconvenience for patients and families, it may improve antibiotic selection and reduce the emergence of resistant strains, which could ultimately preserve future treatment options. In addition, children are frequently exposed to antibiotics for common childhood infections, which may further contribute to resistance. Data regarding prior antibiotic exposure for non-*H. pylori* indications were not consistently available in our cohort and may have influenced the observed resistance patterns. This highlights the importance of developing standardized templates within electronic medical records to improve documentation of prior antibiotic exposure, as well as supporting initiatives such as the creation of local, regional, or global registries.

Limitations of this study include the retrospective design and tertiary care setting, which may introduce selection bias and limit generalizability to broader pediatric populations. The sample size, while including a relatively robust representation of Black and Asian patients, may be insufficient to detect smaller effect sizes. The relatively small number of resistant isolates for certain antibiotics, particularly amoxicillin and tetracycline, limited the ability to perform multivariable analyses for these outcomes and may have reduced the precision and stability of some estimates. Treatment regimens, eradication strategies, and eradication outcomes were not evaluated in the present study, as these aspects have been previously reported by our group in a subset of this cohort, corroborating prior evidence that antimicrobial resistance negatively impacts eradication outcomes. Additionally, data on prior antibiotic exposure for non-*H. pylori* indications were not available, which may represent an unmeasured confounder in the associations between demographic factors and antimicrobial resistance. Finally, race and ethnicity should be interpreted as proxies for social, geographic, and exposure-related factors rather than biological determinants of resistance.

## 5. Conclusions

High rates of *H. pylori* resistance to metronidazole and clarithromycin, exceeding the 15% threshold recommended to limit empiric use, were observed in this pediatric cohort. We also identified a notable rate of rifampin resistance (12.7%), a concerning finding given that rifampin has been proposed as an alternative option in adult treatment regimens, and therapeutic options for children remain limited. These findings highlight the importance of careful clinical decision-making when considering treatment for pediatric *H. pylori* infection, including thoughtful assessment of prior treatment history, demographic and epidemiologic factors, and the overall risks and benefits of eradication therapy. In settings with high resistance rates and limited empiric options, susceptibility-guided therapy may be particularly important to optimize treatment success while minimizing unnecessary antibiotic exposure and the potential for further resistance development. Our study adds to the growing literature demonstrating the complexity of antimicrobial resistance patterns in pediatric *H. pylori* infection and stresses the need for expanded access to antimicrobial susceptibility testing in clinical practice. Larger multicenter studies including children from diverse geographic regions are needed to confirm these findings and further define risk factors associated with antimicrobial resistance.

## Figures and Tables

**Table 1:** Demographic and Clinical Characteristics.

Characteristic	*n* = 174 ^1^
Age (years)	14.0 (9.0–16.0)
Age group	
2–5	16 (9.2%)
6–11	43 (24.9%)
12–17	90 (52.0%)
18–21	24 (13.9%)
Sex	
Male	97 (56.1%)
Female	76 (43.9%)
Race	
White	70 (40.5%)
Black or African American	38 (22.0%)
Asian	34 (19.7%)
Other	31 (17.9%)
Hispanic ethnicity	67 (38.7%)
Prior *H. pylori* treatment	20 (11.6%)
Antibiotic resistance	
Clarithromycin (CLA)	32 (18.5%)
Metronidazole (MTZ)	49 (28.3%)
Fluoroquinolone (FLQ)	20 (11.5%)
Rifampin (RIF)	22 (12.7%)
Amoxicillin (AMX)	7 (4.0%)
Tetracycline (TET)	1 (0.6%)
Multi-drug resistance (≥2)	30 (17.3%)

^1^ Median (Q1–Q3); *n* (%).

**Table 2:** Antibiotic Resistance Prevalence by Demographic Characteristics.

	Resistance Prevalence (%) and *p*-Value					
Characteristic	CLA	MTZ	AMX	FLQ	TET	RIF
Sex	ns	ns	ns	ns	ns	ns
Male	22.7%	33%	5.2%	15.5%	0%	9.3%
Female	13.2%	22.4%	2.6%	6.6%	1.3%	17.1%
Race	ns	**<0.05**	**<0.01**	ns	ns	ns
White	18.6%	20%	0%	12.9%	0%	14.3%
Black or African American	10.5%	44.7%	2.6%	15.8%	0%	15.8%
Asian	32.4%	23.5%	14.7%	5.9%	0%	2.9%
Other	12.9%	32.3%	3.2%	9.7%	3.2%	16.1%
Hispanic ethnicity	ns	ns	**<0.05**	ns	ns	ns
No	18.9%	24.5%	6.6%	10.4%	0%	11.3%
Yes	17.9%	34.3%	0%	13.4%	1.5%	14.9%
Age group	**<0.01**	ns	ns	ns	ns	ns
2–5	37.5%	12.5%	0%	12.5%	6.2%	0%
6–11	30.2%	25.6%	7%	11.6%	0%	18.6%
12–17	12.2%	33.3%	3.3%	7.8%	0%	13.3%
18–21	8.3%	25%	4.2%	25%	0%	8.3%
Prior treatment	**<0.001**	**<0.001**	ns	**<0.05**	ns	ns
No	13.7%	23.5%	3.9%	8.5%	0.7%	12.4%
Yes	55%	65%	5%	35%	0%	15%

Fisher’s exact test *p*-values shown in header rows. Bold *p*-values = *p* < 0.05. ns = non-significant *p*-value. % = proportion of resistant isolates within each demographic subgroup.

**Table 3:** Multivariable Logistic Regression—Predictors of Antibiotic Resistance.

			OR (95% CI) and *p*-Value					
Characteristic	CLA		MTZ		FLQ		RIF	
Age (years)	0.88 (0.80–0.97)	**<0.05**	1.02 (0.94–1.11)	ns	1.03 (0.92–1.16)	ns	0.97 (0.87–1.08)	ns
Sex								
Male	—		—		—		—	
Female	0.58 (0.23–1.43)	ns	0.70 (0.33–1.47)	ns	0.47 (0.14–1.35)	ns	2.33 (0.91–6.26)	ns
Race								
White	—		—		—		—	
Black or African American	0.58 (0.13–2.14)	ns	5.73 (2.08–17.1)	**0.001**	1.39 (0.36–5.17)	ns	1.23 (0.36–3.98)	ns
Asian	1.55 (0.46–5.18)	ns	2.06 (0.61–6.92)	ns	0.46 (0.06–2.50)	ns	0.16 (0.01–1.01)	ns
Other	0.63 (0.15–2.24)	ns	2.14 (0.75–6.11)	ns	0.77 (0.15–3.05)	ns	1.26 (0.35–4.15)	ns
Hispanic ethnicity								
No	—		—		—		—	
Yes	0.99 (0.34–2.86)	ns	2.45 (1.03–6.12)	**<0.05**	1.19 (0.37–3.90)	ns	1.07 (0.38–3.01)	ns
Prior treatment								
No	—		—		—		—	
Yes	7.58 (2.63–23.1)	**<0.001**	6.85 (2.42–21.2)	**<0.001**	5.36 (1.66–16.9)	**<0.01**	1.70 (0.35–6.38)	ns

OR = Odds Ratio; CI = Confidence Interval. Reference categories: Sex = Male; Race = White; Hispanic ethnicity = No; Prior treatment = No. Amoxicillin and tetracycline were excluded due to insufficient number of resistant cases (*n* = 7 and *n* = 1, respectively). All models were adjusted for age, sex, race, Hispanic ethnicity, and prior *H. pylori* treatment.

## Data Availability

The original contributions presented in this study are included in the article. Further inquiries can be directed to the corresponding authors.

## Supplementary Materials

The following supporting information can be downloaded at https://www.mdpi.com/article/10.3390/pathogens15060608/s1, Figure S1: Co-resistance correlation matrix among *H. pylori* antibiotic isolates.

## Abbreviations

The following abbreviations are used in this manuscript:

CLA	Clarithromycin
TET	Tetracycline
AMX	Amoxicillin
MTZ	Metronidazole
FLQ	Fluoroquinolone
RIF	Rifampin

## References

[1] Li Y., Choi H., Leung K., Jiang F., Graham D.Y., Leung W.K. (2023). Global Prevalence of *Helicobacter pylori* Infection between 1980 and 2022: A Systematic Review and Meta-Analysis. Lancet Gastroenterol. Hepatol..

[2] Rugge M., Genta R.M., Malfertheiner P., Dinis-Ribeiro M., El-Serag H., Graham D.Y., Kuipers E.J., Leung W.K., Park J.Y., Rokkas T. (2024). RE.GA.IN.: The Real-World Gastritis Initiative-Updating the Updates. Gut.

[3] Malfertheiner P., Megraud F., Rokkas T., Gisbert J.P., Liou J.-M., Schulz C., Gasbarrini A., Hunt R.H., Leja M., O’Morain C. (2022). Management of *Helicobacter pylori* Infection: The Maastricht VI/Florence Consensus Report. Gut.

[4] Chey W.D., Howden C.W., Moss S.F., Morgan D.R., Greer K.B., Grover S., Shah S.C. (2024). ACG Clinical Guideline: Treatment of *Helicobacter pylori* Infection. Am. J. Gastroenterol..

[5] Homan M., Jones N.L., Bontems P., Carroll M.W., Czinn S.J., Gold B.D., Goodman K., Harris P.R., Jerris R., Kalach N. (2024). Updated Joint ESPGHAN/NASPGHAN Guidelines for Management of *Helicobacter pylori* Infection in Children and Adolescents (2023). J. Pediatr. Gastroenterol. Nutr..

[6] Mégraud F., Graham D.Y., Howden C.W., Trevino E., Weissfeld A., Hunt B., Smith N., Leifke E., Chey W.D. (2023). Rates of Antimicrobial Resistance in *Helicobacter pylori* Isolates from Clinical Trial Patients Across the US and Europe. Am. J. Gastroenterol..

[7] Savoldi A., Carrara E., Graham D.Y., Conti M., Tacconelli E. (2018). Prevalence of Antibiotic Resistance in *Helicobacter pylori*: A Systematic Review and Meta-Analysis in World Health Organization Regions. Gastroenterology.

[8] Salahi-Niri A., Nabavi-Rad A., Monaghan T.M., Rokkas T., Doulberis M., Sadeghi A., Zali M.R., Yamaoka Y., Tacconelli E., Yadegar A. (2024). Global Prevalence of *Helicobacter pylori* Antibiotic Resistance among Children in the World Health Organization Regions between 2000 and 2023: A Systematic Review and Meta-Analysis. BMC Med..

[9] Kato S., Shimizu T., Toyoda S., Gold B.D., Ida S., Ishige T., Fujimura S., Kamiya S., Konno M., Kuwabara K. (2020). The Updated JSPGHAN Guidelines for the Management of *Helicobacter pylori* Infection in Childhood. Pediatr. Int..

[10] Harris P.R., Lucero Y., Pierre R. (2021). Adaptation to the Reality of Latin America of the NASPGHAN/ESPGHAN 2016 Guidelines on the Diagnosis, Prevention, and Treatment of *Helicobacter pylori* Infection in Pediatrics. J. Pediatr. Gastroenterol. Nutr..

[11] Arellanos K.R., Cardini J., Joerger J., Estrella-Pimentel L., Goldsmith J.D., Bonilla S. (2026). Standardizing a Protocol for *Helicobacter pylori* Gastric Biopsy Culture: From Implementation to Sustained Practice. JPGN Rep..

[12] Megraud F., Bruyndonckx R., Coenen S., Wittkop L., Huang T.-D., Hoebeke M., Bénéjat L., Lehours P., Goossens H., Glupczynski Y. (2021). *Helicobacter pylori* Resistance to Antibiotics in Europe in 2018 and Its Relationship to Antibiotic Consumption in the Community. Gut.

[13] Kori M., Le Thi T.G., Werkstetter K., Sustmann A., Bontems P., Lopes A.I., Oleastro M., Iwanczak B., Kalach N., Misak Z. (2020). *Helicobacter pylori* Infection in Pediatric Patients Living in Europe: Results of the EuroPedHP Registry 2013 to 2016. J. Pediatr. Gastroenterol. Nutr..

[14] Duck W.M., Sobel J., Pruckler J.M., Song Q., Swerdlow D., Friedman C., Sulka A., Swaminathan B., Taylor T., Hoekstra M. (2004). Antimicrobial Resistance Incidence and Risk Factors among *Helicobacter pylori*–Infected Persons, United States. Emerg. Infect. Dis..

[15] Mosites E., Bruden D., Morris J., Reasonover A., Rudolph K., Hurlburt D., Hennessy T., McMahon B., Bruce M. (2018). Antimicrobial Resistance among *Helicobacter pylori* Isolates in Alaska, 2000–2016. J. Glob. Antimicrob. Resist..

[16] White B., Winte M., DeSipio J., Phadtare S. (2022). Clinical Factors Implicated in Antibiotic Resistance in *Helicobacter pylori* Patients. Microorganisms.

[17] Tveit A.H., Bruce M.G., Bruden D.L., Morris J., Reasonover A., Hurlburt D.A., Hennessy T.W., McMahon B. (2011). Alaska Sentinel Surveillance Study of *Helicobacter pylori* Isolates from Alaska Native Persons from 2000 to 2008. J. Clin. Microbiol..

[18] Huang J.G., Lim S.Y.S., Aw M.M., Quak S.-H. (2022). Antibiotic Resistance Patterns and Therapeutic Outcomes of Pediatric *Helicobacter pylori* Infection in a High-Migrant Singaporean Cohort. Helicobacter.

[19] Kori M., Yahav J., Berdinstein R., Shmuely H. (2017). Primary and Secondary Antibiotic Resistance of *Helicobacter pylori* in Israeli Children and Adolescents. Isr. Med. Assoc. J..

[20] Harrison U., Fowora M.A., Seriki A.T., Loell E., Mueller S., Ugo-Ijeh M., Onyekwere C.A., Lesi O.A., Otegbayo J.A., Akere A. (2017). *Helicobacter pylori* Strains from a Nigerian Cohort Show Divergent Antibiotic Resistance Rates and a Uniform Pathogenicity Profile. PLoS ONE.

[21] Riaz M., Chan C., Andrews C., Herzlinger M., Liu E., Bonilla S. (2025). Factors Associated with *Helicobacter pylori* Antimicrobial Resistance in a US Pediatric Cohort. J. Pediatr. Gastroenterol. Nutr..

[22] HELIS Overview: *Helicobacter pylori*. Culture with Antimicrobial Susceptibilities, Varies. https://www.mayocliniclabs.com/test-catalog/overview/62769.

[23] U.S. Census Bureau About the Topic of Race. https://www.census.gov/topics/population/race/about.html.

[24] Le Thi T.G., Werkstetter K., Kotilea K., Bontems P., Cabral J., Cilleruelo Pascual M.L., Kori M., Barrio J., Homan M., Kalach N. (2023). Management of *Helicobacter pylori* Infection in Paediatric Patients in Europe: Results from the EuroPedHp Registry. Infection.

[25] Bonilla S., Mitchell P.D., Mansuri I. (2021). Low Adherence to Society Guidelines for the Management of *Helicobacter pylori* Among Pediatric Gastroenterologists. J. Pediatr. Gastroenterol. Nutr..

[26] Xu L., Li X.-T., Ur-Rahman I., Zhang C., Qi Y.-B., Hu R.-B., Li K., Awadh A.M., Ma J., Xiao W. (2024). Global *H. pylori* Recurrence, Recrudescence, and Re-Infection Status after Successful Eradication in Pediatric Patients: A Systematic Review and Meta-Analysis. J. Gastroenterol..

[27] Talarico S., Korson A.S., Leverich C.K., Park S., Jalikis F.G., Upton M.P., Broussard E., Salama N.R. (2018). High Prevalence of *Helicobacter pylori* Clarithromycin Resistance Mutations among Seattle Patients Measured by Droplet Digital PCR. Helicobacter.

[28] Guo S., Sun Q., Zhao X., Shen L., Zhen X. (2021). Prevalence and Risk Factors for Antibiotic Utilization in Chinese Children. BMC Pediatr..

[29] Zhang J., Ma X., Tang L., Tian D., Lin L., Li Y., Lu G., Si L., Zhang W., Qian J. (2022). Pattern of Antibiotic Prescriptions in Chinese Children, A Cross-Sectional Survey From 17 Hospitals Located Across 10 Provinces of China. Front. Pediatr..

[30] Nguyen N.V., Do N.T.T., Vu D.T.V., Greer R.C., Dittrich S., Vandendorpe M., Pham T.N., Ta N.T.D., Pham T.Q., Khuong V.T. (2023). Outpatient Antibiotic Prescribing for Acute Respiratory Infections in Vietnamese Primary Care Settings by the WHO AWaRe (Access, Watch and Reserve) Classification: An Analysis Using Routinely Collected Electronic Prescription Data. Lancet Reg. Health West. Pac..

[31] Bert F., Previti C., Calabrese F., Scaioli G., Siliquini R. (2022). Antibiotics Self Medication among Children: A Systematic Review. Antibiotics.

[32] Zhao P., Zhao J., Shi H., Meng F., Yang N., Dong L., Gong J. (2023). Relationship between Antibiotic Resistance and the cagA and vacA Genotypes among *Helicobacter pylori* Strain Isolates from Patients in Xi’an. Braz. J. Microbiol..

