# Supplementary material for: Predictors of Resistance in Pediatric Helicobacter pylori Infection
Source: Pathogens. 2026 Jun 5;15(6):608. doi: 10.3390/pathogens15060608 (PMC13304559; doi:10.3390/pathogens15060608)
Supplement: Supplementary file 1 [file pathogens-15-00608-s001.zip › pathogens-4295881-supplementary.pdf]

Supplementary Materials

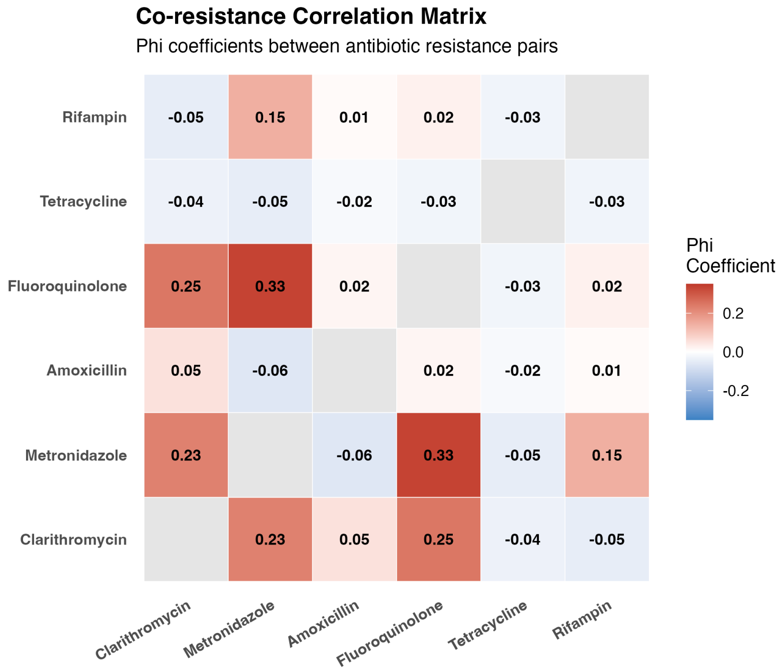

Figure S1. Co-resistance correlation matrix among *H. pylori* antibiotic isolates. Values represent Phi coefficients, a measure of association between binary resistance variables. All pairwise coefficients were below 0.35, indicating weak correlations between resistances to different antimicrobials. The strongest associations were observed between fluoroquinolone and metronidazole (φ = 0.33) and clarithromycin and fluoroquinolone (φ = 0.25). Grey cells indicate self-correlations (φ = 1.0). *n* = 174 pediatric patients.
